# Study of Leaf Metabolome Modifications Induced by UV-C Radiations in Representative *Vitis*, *Cissus* and *Cannabis* Species by LC-MS Based Metabolomics and Antioxidant Assays

**DOI:** 10.3390/molecules190914004

**Published:** 2014-09-05

**Authors:** Guillaume Marti, Sylvain Schnee, Yannis Andrey, Claudia Simoes-Pires, Pierre-Alain Carrupt, Jean-Luc Wolfender, Katia Gindro

**Affiliations:** 1 School of Pharmaceutical Sciences, EPGL, University of Geneva, University of Lausanne, Quai Ernest-Ansermet 30, Geneva CH-1211, Switzerland; E-Mails: yannis.andrey@gmail.com (Y.A.); claudia.avello@unige.ch (C.S.-P.); pierre-alain.carrupt@unige.ch (P.-A.C.); jean-luc.wolfender@unige.ch (J.-L.W.); 2 Station de recherche Agroscope, Institut des Sciences en Production Végétale IPV, Route de Duiller 50, P.O. Box 1012, Nyon 1260, Switzerland; E-Mails: sylvain.schnee@agroscope.admin.ch (S.S.); katia.gindro@agroscope.admin.ch (K.G.)

**Keywords:** phytoalexins, UV-C stress, metabolomics, LC-MS, *Cannabis sativa*, *Cissus antarctica*, *Vitis vinifera*, chemodiversity

## Abstract

UV-C radiation is known to induce metabolic modifications in plants, particularly to secondary metabolite biosynthesis. To assess these modifications from a global and untargeted perspective, the effects of the UV-C radiation of the leaves of three different model plant species, *Cissus antarctica* Vent*.* (Vitaceae), *Vitis vinifera* L. (Vitaceae) and *Cannabis sativa* L. (Cannabaceae), were evaluated by an LC-HRMS-based metabolomic approach. The approach enabled the detection of significant metabolite modifications in the three species studied. For all species, clear modifications of phenylpropanoid metabolism were detected that led to an increased level of stilbene derivatives. Interestingly, resveratrol and piceid levels were strongly induced by the UV-C treatment of *C. antarctica* leaves. In contrast, both flavonoids and stilbene polymers were upregulated in UV-C-treated *Vitis* leaves. In *Cannabis*, important changes in cinnamic acid amides and stilbene-related compounds were also detected. Overall, our results highlighted phytoalexin induction upon UV-C radiation. To evaluate whether UV-C stress radiation could enhance the biosynthesis of bioactive compounds, the antioxidant activity of extracts from control and UV-C-treated leaves was measured. The results showed increased antioxidant activity in UV-C-treated *V. vinifera* extracts.

## 1. Introduction

Naturally occurring compounds play an essential role in drug discovery. From 1981 to 2010, 64% of new approved therapeutic agents were inspired or directly derived from natural products [1,2]. The exploration of natural biodiversity has led to the identification of a remarkable variety of chemical entities that possess highly selective and specific biological activities and unique modes of action [3]. Bioprospecting of natural sources is still of great interest for the discovery of new scaffolds; only 1% of tropical species have been investigated for their biological activities [4]. Another aspect increasing the potential of bioresources is the ability of organisms to respond to biotic and abiotic stress by inducing biosynthetic pathways that create an array of secondary metabolites not otherwise detected in steady-state conditions [5]. Recent advances in genomics have highlighted gene clusters that remain silent in the absence of a specific trigger [6]. This hidden chemodiversity has been primarily explored in microorganisms through growth media alterations, and various stresses and genetic manipulations to unlock overlooked biosynthetic pathways and thus provide new metabolic diversity [7]. Some studies have also uncovered the ability of plants to enhance the biosynthesis of bioactive compounds upon stress or stimuli, thus improving their chemical defences, which in turn can be exploited to generate new stress-induced chemical entities of potential therapeutic value [8]. For example, the exposure of the roots of hydroponically grown plants to certain chemical agents induced the production of bioactive compounds, and the corresponding crude extracts were twice as likely to have *in vitro* activity against bacteria, fungi, or cancer in screening programs [9]. Abiotic stresses, such as ultraviolet (UV) radiation, are also known to stimulate plant defences and efficiently increase resistance to pathogens [10]. Phytochemical investigations of plant responses to UV stress have revealed the induction of phenolics such as stilbenoids in various *Vitis* sp., presumably antioxidants, that may protect cells against UV-induced oxidative damage [11,12]. Interestingly, other classes of secondary metabolites were also up-regulated, such as sesquiterpenes (rishitin) in tomato fruits [13], or phenylamides in rice leaves [14], and the benzolactam derivative wasalexins in the leaves of Salt cress (*Thellungiella halophila*) [15] or alkaloid derivatives like brachycerine in *Psychotria brachyceras* [16]. These few examples demonstrate the ability of UV radiation to induce phytoalexin biosynthesis, thus improving the chemical diversity of treated extracts, which in turn could improve their effects on given biological targets [17]. A recent study has shown the contrasting effects of UV-C irradiation of the leaves of several plant species on their antifungal activities. Interestingly, of the eighteen species tested, five species demonstrated a net increase of antifungal properties against a clinical *Fusarium solani* strain, whereas the activity of three extracts was decreased [18]. While most of the studies have focused on the biosynthesis of some characteristic phytoalexins, global metabolome perturbations caused by this intense abiotic stress have not yet been studied. Metabolomics provides a holistic overview of the global changes occurring after stress, chemical induction or genetic manipulation [19]. In particular, untargeted liquid chromatography-mass spectrometry (LC-MS)-based approaches are well suited to reveal the effects of biotic or abiotic stresses in plants at both primary and secondary metabolite levels. As an example, this approach has been used to assess the metabolic response of maize leaves after infestation by *Spodoptera frugiperda* larvae [20]. To evaluate metabolic changes upon UV treatment in leaves, three species known to produce a variety of phenolic secondary metabolites were chosen. *Vitis vinifera* leaves were first analysed, because stilbene biosynthesis upon abiotic or biotic stresses is well documented for this genus [21,22]. As with *V. vinifera*, the genus *Cissus* belongs to the Vitaceae family and is also known to produce various stilbenoids [23]. Several secondary metabolites have been isolated from *C. sativa*, including cannabinoids, flavonoids, alkaloids and stilbenoids, with characteristic structural backbones such as spirans, phenanthrenes and bibenzyls [24]. The goal of this study was to assess if leaves response to UV-C radiation could induce new chemical entities able to improve the antioxidant activity of the crude extract. Firstly, a differential LC-MS-based metabolomics approach was used to provide an overview of all the UV-induced modifications of the chemical compositions of the extracts studied. Several biomarkers, including characteristic phytoalexins of each species, were putatively identified based on molecular formula assignment from the high-resolution MS (HRMS) data recorded. Secondly, variations in the antioxidant activity were used to obtain an initial indication of the UV-C-induced chemical changes in a crude extract. Radical-scavenging activity was assessed with both DPPH and ABTS assays, and antioxidant activity was assessed with the oxygen radical absorbance capacity (ORAC) assay.

## 2. Results and Discussion

### 2.1. LC-MS-Based Metabolomics Approach

**Figure 1:**
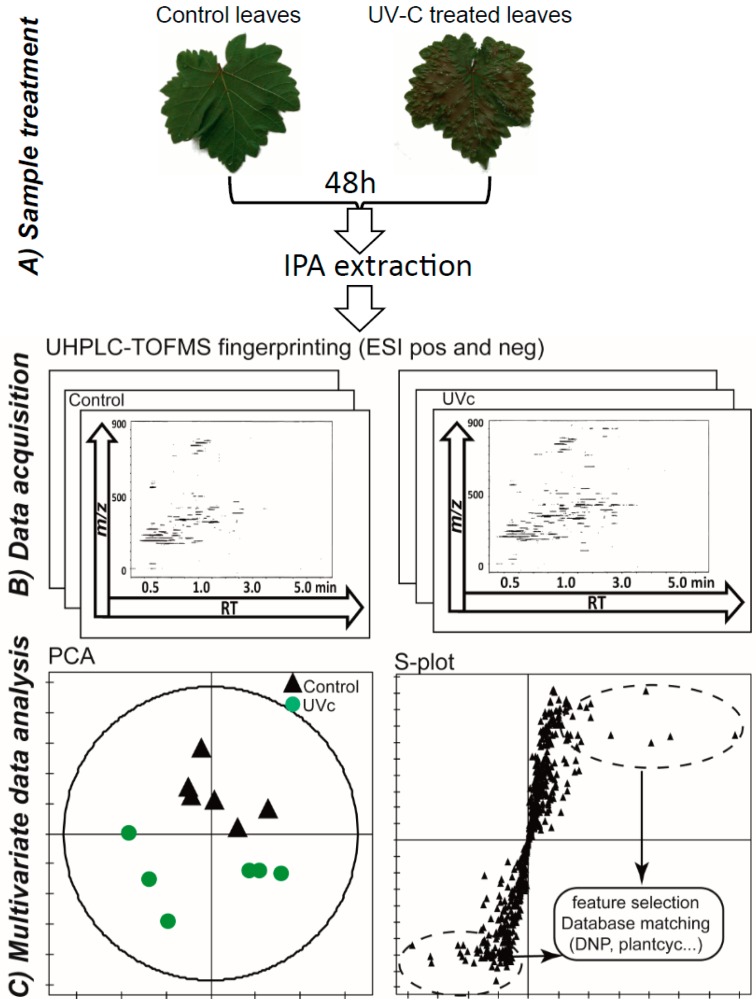
UHPLC-TOFMS metabolomics approach: (**A**) Leaves from independent plantlets were irradiated with UV-C and then extracted using IPA (isopropanol) after 48 h. Pictures of *Vitis vinifera* leaves are displayed. As shown, morphological changes can already be observed after 48 h (**B**) 2D ion maps of NI ESI UHPLC-TOFMS rapid metabolite profiling for each extract; (**C**) Principal component analysis (PCA) of *V. vinifera* LC-MS data in NI mode and S-plot display after OPLS-DA for the selection of the most important features for further annotation.

Preliminary experiments of UV-C radiation on entire plants and detached leaves of *Vitis vinifera* have shown a similar induction of stilbenoid polymers [12,25]. However, UV-C radiation of entire plants induced a strong water stress within a few hours and displayed poor reproducibility between biological replicates. Thus, fresh leaves of *Vitis vinifera*, *Cissus antarctica* and *Cannabis sativa* were harvested from different plantlets and exposed to UV-C radiation for 10 min since an extended period of exposition to UV will trigger drastic and irremediable damages to tissue integrity. Seven biological replicates per case were profiled by reversed-phase ultra high performance liquid chromatography-time of flight mass spectrometry (UHPLC-TOFMS) using a generic MeCN-H_2_O gradient after simple sample preparation on solid phase extraction (SPE) to remove pigments and very apolar compounds [26]. The LC-MS data, recorded in both positive (PI) and negative (NI) electrospray ESI ionisation modes, were processed using MZmine 2.10 to extract features characterised by their *m/z* ratio, retention time and area. Then, principal component analysis (PCA) was used as an exploratory step prior to multivariate supervised analysis (Figure 1B).

To evaluate rapidly when major modifications occur, a few leaves were analysed 24, 48 and 72 h after UV-C exposure. The quenching and extraction of leaves 48 hours after UV-C radiation revealed the most significant metabolomic variations. Differences between control and treated leaves were less pronounced when sampling was performed after 24 h. However, 72 h after radiation, degradation of the leaf surface was noticeable at many locations, and the variation between biological replicates was significant (data not shown). Thus, a single time point 48 h after UV-C treatment was chosen, because a reproducible and reliable metabolic response was observed without apparent material degradation (Figure 1A).

After PCA, a discriminant analysis approach (orthogonal projections to latent structures-discriminant analysis, OPLS-DA) was applied to obtain classification models and to highlight putative features involved in the stress response according to their variable important projection (VIP) values and position on an S-plot (Figure 1C) [27].

### 2.2. Data Treatment and Analysis

**Figure 2:**
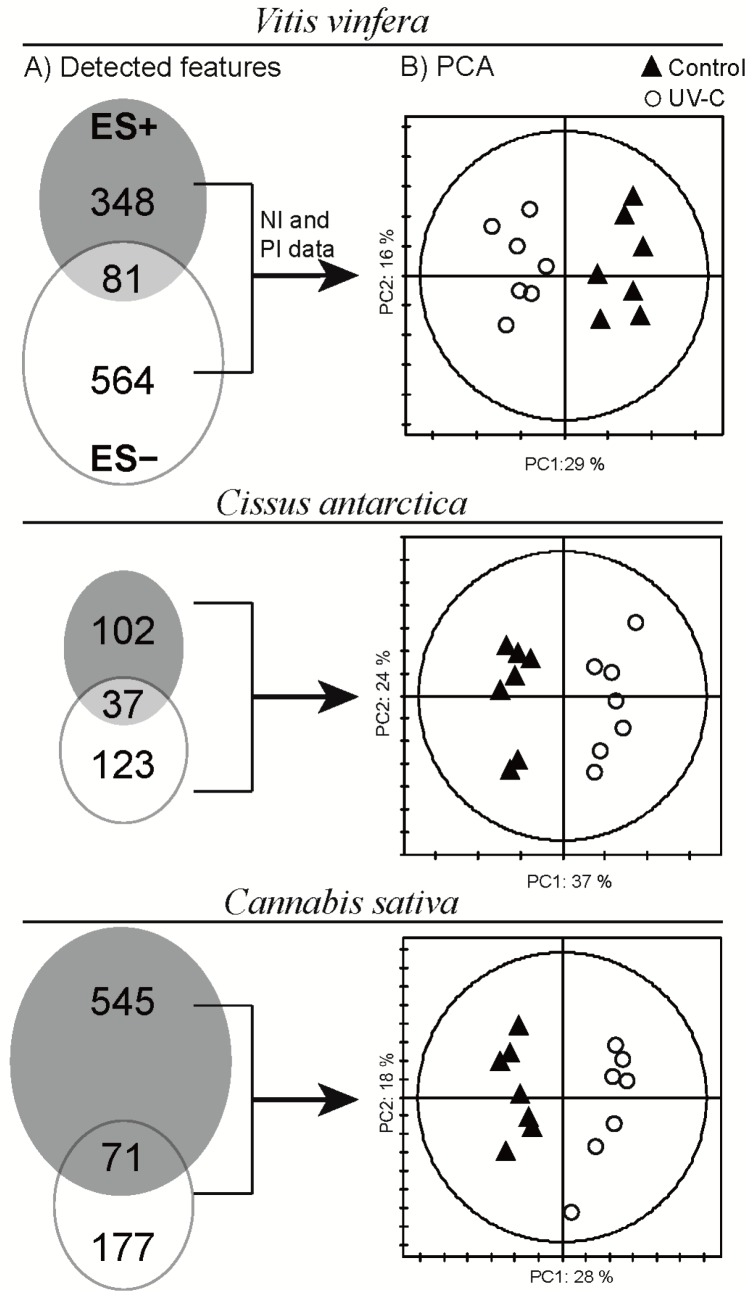
(**A**) Venn diagram showing the distribution of detected features in PI (grey circle) and NI (white circle) ESI UHPLC-TOFMS modes. The intercept shows the number of features detected in both modes; (**B**) Principal component analysis of concatenated PI and NI datasets scaled in unit variance.

To evaluate LC-MS data, the PI and NI datasets were compared (RT: ±0.2 min, *m/z*: ±10 ppm) after removing features identified as adducts or ion complexes (Venn diagram, Figure 2A). The number of features detected varied significantly according to the species studied and the ionisation mode used. It was interesting that only 8% to 12% of detected features were common to PI and NI modes, thus emphasising the complementary information provided by both ionisation processes (Figure 2A). To incorporate all these data, the PI and NI datasets from each species were concatenated, and multivariate data analysis (low level data fusion) was performed [28,29]. Unit variance scaling was used to reduce the effect of differential sensitivity between the ionisation modes. Overall, PCA displayed two well-separated clusters corresponding to control and UV-C-treated leaves along the first principal component for the three species studied (Figure 2B). This result indicated that the leaves of all three species react strongly to UV-C treatment at the metabolome level.

### 2.3. Global Estimation of the Leaf Metabolome Modifications upon UV-C Treatment

Following PCA, LC-MS datasets underwent OPLS-DA. A 7-fold procedure was used to cross-validate discriminant models of each species (*V. vinifera* model: R2Y = 0.98, Q2Y = 0.92; *C. Antarctica* model: R2Y = 0.99, Q2Y = 0.96; *C. sativa* model: R2Y = 0.99, Q2Y = 0.96). The VIP scores were used to rank the features according to their contribution to the discriminant model. Only VIP scores greater than one were retained for further analysis, because they are the most relevant for explaining the Y response (*i.e.*, UV-C leaf treatment response) [30]. Then, significant features were subjected to an unpaired T-test (α = 0.05), and only fold changes greater than five between control and UV-C treated samples were retained for further analysis. Thus, a combination of multivariate and univariate analyses was used to estimate the number of up- and down-regulated features in each species.

**Figure 3:**
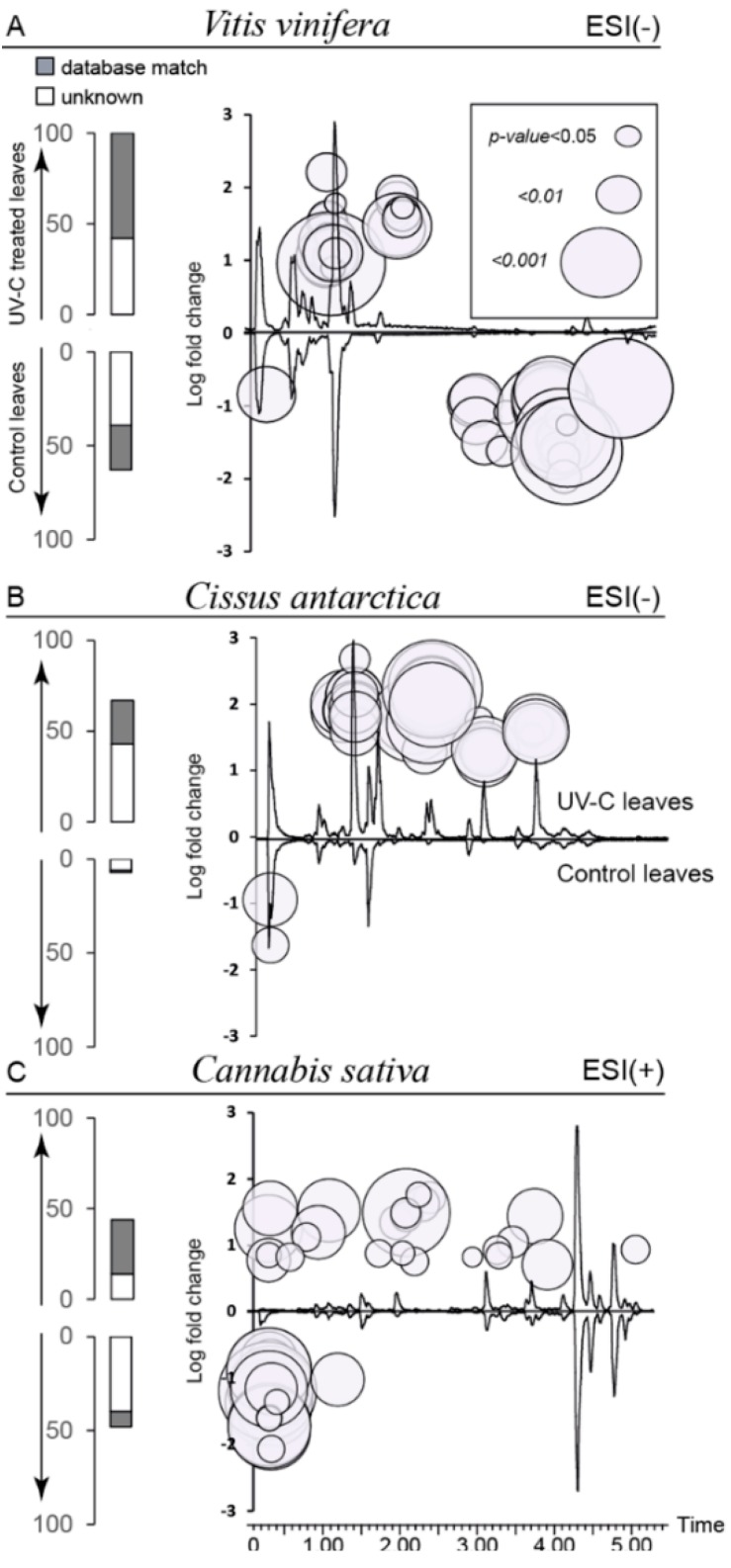
**Right**: LC-MS chromatogram of control (BPI trace) (**bottom**) and UV-C-treated leaves (**top**). Superimposed are the main features plotted according to their mean log-fold change between control and UV-C treatment. The area of the circle is inversely proportional to the *p*-value of an unpaired T-test, α = 0.05. **Left**: bar plot showing the number of features up- and down-regulated in UV-treated leaves (>5-fold intensity changes). Blank bar shows unidentified features; grey bar shows features with a database match.

In *V. vinifera* leaves, 100 features were significantly up-regulated upon UV-C radiation, and 55 were down-regulated (bar plot, Figure 3A). This high number corresponds to approximately 15% of the total number of features detected. In *C. antarctica* leaves, 60 features were up-regulated and three were significantly down-regulated, which represents 23% of total features detected in this species (bar plot, Figure 3B). In the case of *C. sativa*, 12% of the total features responded significantly, with 48 features elicited and 49 down-regulated (bar plot, Figure 3C).

To provide an overview of the major changes observed, all significant features were superimposed on short LC-MS profiles of each species (Figure 3, UV-C-treated upper trace and control bottom trace). The features were positioned according to their log-fold changes (Y) and their retention times (X), with point sizes calculated from unpaired T-test *p*-values on the bubble chart. In *V. vinifera* (Figure 3A), the bubble chart clearly show that major up-regulated features are moderately polar and eluted between 1 and 2 min, whereas most of the down-regulated features had longer retention times (less polar metabolites). Interestingly, in the case of *C. antarctica* (Figure 3B), most of the induced features appeared in the same area as for *V. vinifera*, but the down-regulated features were detected close to the injection peak. The up-regulated features of *C. sativa* were distributed all along the chromatogram, whereas the down-regulated ones eluted close to the injection peak (Figure 3C). These charts display the response specificity of each species and give an idea of the polarity of significant features.

### 2.4. Biomarkers of UV-C Radiation

Significant features were annotated based on accurate HRMS exact mass spectral data and the use of heuristic filters [31] after deconvolution and adduct removal. Molecular formula determination and further cross-searching based on chemotaxonomy information were then performed. Putative identification was achieved using the molecular formula together with the botanical genus and family as queries in the Dictionary of Natural Products database [32]. Unidentified metabolites were then matched using Lipidmaps [33] and the Plant Metabolic Network [34], generic databases related to plant metabolism. These labels correspond to level 2 and 3 IDs according to the Metabolite Identification Task Group [35]. Altogether, the putative identification of approximately 50% of the significant features (>5-fold changes) in each species was achieved (bar plots, Figure 3).

In the three studied species, several down-regulated metabolites were detected close to the injection peak (possibly polar or charged metabolites) (bubble charts, Figure 3). Feature annotation highlighted that several simple organic acids, such as malic acid and fumaric acid, could be down-regulated in *V. vinifera* and *C. antarctica* leaves. In addition, a few phosphorylated metabolites, such as adenosine diphosphate and O-phospho-L-homoserine, which are involved in methionine biosynthesis, were down-regulated in *V. vinifera*. In *C. sativa*, α-iminosuccinate, which is involved in cofactor biosynthesis, and N-acetylglutamyl phosphate were also down-regulated (data not shown). A detailed identification of such polar metabolites would require the use of alternative profiling methods that focus on primary metabolites, such as hydrophilic interaction liquid chromatography (HILIC) [36] or gas chromatography-mass spectrometry (GC-MS) [37]. However, this pursuit was beyond the scope of this study, which is primarily dedicated to secondary metabolite induction.

Late-eluting metabolites (after 3 min) were putatively identified as glycerophospholipids (GPLs). Several GPLs were detected in reduced quantities in UV-C treated *V. vinifera* leaves compared to control. As major constituents of plant cell membranes, GPLs are particularly sensitive to denaturation upon UV radiation. This behaviour is mainly due to the oxidative damage by reactive oxygen species (ROS) of the methylene groups in unsaturated fatty acids, which leads to a chain reaction of peroxidation [10,38]. Interestingly, GPLs were induced in *C. antarctica* and *C. sativa* UV-C treated leaves (Table 1). Damages caused by UV-C do not involve specific cellular receptors but stimulate a metabolic response similar to that caused by wounding [39]. For instance, a systemic induction of phosphatidic acid and lysophospholipids was found in wounded tomato leaves [40]. The possible connection of phospholipids to jasmonates is illustrated by the fact that silencing phospholipase D in rice limits the induction of jasmonic acid levels [41]. Although the untargeted LC-MS profiling conducted here did not reveal any induction of jasmonates, our data reveal some evidence of cell membrane reconfiguration upon UV-C radiation.

**Table 1:** Putative identification of induced compounds in UV-C treated leaves.

Mode	HR-MS	RT (min.)	MF	Chemical Class	Database (hit) ^a^	Putative ID ^b^	Error (mDa)	Isotope Pattern Score (%)	Fold Change (UV/C)
*Vitis vinifera* L.									
**NI**	405.1178	1.22	C_20_H_22_O_9_	stilbene	Lipidmaps (5)	astringin	1.2	95	150
**NI**	453.1327	1.41	C_28_H_22_O_6_	stilbene polymer	DNP (6)	ε-viniferin	1.6	97	120
**NI**	919.2451	1.74	C_56_H_40_O_13_	stilbene polymer	DNP (1)	amurensin K	0.3	96	110
**NI**	471.1455	1.47	C_28_H_24_O_7_	stilbene polymer	DNP (1)	amurensin A	0.6	95	90
**NI**	679.2027	1.88	C_42_H_32_O_9_	stilbene polymer	DNP (6)	vitisin E	1.2	95	70
**NI**	597.1815	0.88	C_27_H_34_O_15_	Flavonoid	Lipidmaps (2)	Catechin 3-O-rutinoside	3.3	95	60
**NI**	231.1013	1.18	C_28_H_22_O_7_	stilbene polymer	DNP (1)	ampelopsin A	2	95	40
**NI**	227.0710	1.62	C_14_H_12_O_3_	stilbene	DNP (1)	resveratrol *	0.2	96	30
*Cissus antarctica* Vent.									
**NI**	227.0709	1.62	C_14_H_12_O_3_	stilbene	DNP (1)	resveratrol *	0.7	98	110
**NI**	435.1295	1.17	C_21_H_24_O_10_	dihydrochalcone flavonoids	DNP (1)	trilobatin	0.1	97	100
**NI**	453.1336	1.58	C_28_H_22_O_6_	stilbene polymer	DNP (2)	pallidol	0.3	95	100
**PI**	637.4055	3.74	C_32_H_61_O_10_P	glycerophospholipids	Lipidmaps (2)	PG(12:0/14:1(9Z))	2.0	95	90
**NI**	389.1229	1.31	C_20_H_22_O_8_	stilbene	DNP (1)	piceid *	1.2	96	40
*Cannabis sativa* L.									
**PI**	259.1348	3.20	C_16_H_18_O_3_	stilbene	Lipidmaps (1)	3-O-methylbatatasin	1.9	95	100
**NI**	407.1881	0.72	C_25_H_28_O_5_	Chalcone flavonoid	DNP (2)	3′-geranyl-2′,4,4′,6′-tetrahydroxychalcone	1.7	95	15
**PI**	625.2543	2.43	C_36_H_36_N_2_O_8_	cinnamic acid amide	DNP (4)	cannabisin D	0.1	96	10
**PI**	235.1697	2.77	C_15_H_22_O_2_	aliphatic	DNP (1)	p-hydroxynonanophenone	0.5	97	8
**PI**	284.1289	1.70	C_17_H_17_NO_3_	cinnamic acid amide	DNP (1)	N-*p*-trans-coumaroyltyramine *	0.8	96	7
**PI**	219.1343	0.49	C_14_H_18_O_2_	spirans	DNP (1)	5,7-dihydroxy[indan-1-spirocyclohexane]	3.0	97	6
**PI**	454.2935	3.97	C_21_H_43_NO_7_P	glycerophospholipids	Lipidmaps (2)	PE(16:0/0:0)	0.7	95	6
**PI**	496.3399	3.98	C_24_H_50_NO_7_P	glycerophospholipids	Lipidmaps (5)	PC(16:0/0:0)	0.1	96	5

^a^ For the DNP database, the molecular formula was crossed-filtered using the genus name or family name. The number of hits is indicated in brackets; ^b^ In the case of more than one hit, the annotation is indicative of a compound characteristic of the class; * Comparison with pure standard. The fold change indicates the ratio of intensity (up-regulation) of a given feature in the UV-C leaves compared to control.

Interestingly, most of the induced metabolites detected in Vitaceae species eluted between 1 and 2 min, denoting compounds with similar physicochemical properties (Figure 3A,B). Following UV-C treatment, several stilbenoids were identified as major up-regulated compounds in *V. vinifera* leaves. The metabolites that were the most significantly induced were putatively annotated as astringin (3-OH-piceid) which has previously been purified from *Vitis* cell cultures [42]. Other strongly induced compounds are stilbene polymers, such as the dehydrodimer ε-viniferin, which was previously detected in UV-C-treated grapevine leaves [12]. Some features putatively identified as glycosylated flavonoids were also up-regulated upon UV-C treatment. As expected, the induction of resveratrol was detected in both *V. vinifera* and *C. antarctica* treated leaves, thus indicating a common genetic background. However, the diversity of stilbene polymers was less pronounced in *C. Antarctica* compared to *V. vinifera*, because only pallidol was detected [43]. Indeed, other phenylpropanoids were identified in this species, such as trilobatin, a dihydrochalcone flavonoid, along with the glycosylated stilbene, piceid. In contrast, *C. sativa* UV-C-treated leaves displayed a different pattern of induction compared to the Vitaceae species studied. Several other classes of induced compounds were putatively identified (GPLs, cinnamic acids, spirans), but the most significantly induced compound was interestingly also found to belong to stilbenes and was putatively identified as 3-hydroxy-5,4'-dimethoxybibenzyl (3-*O*-methylbatatasin). This compound could be biosynthesised from dihydroresveratrol as has already been reported to occur in *C. sativa* [44]. The coupling of dihydrostilbenes followed by reductive steps could also lead to spirans, such as the induced 5,7-dihydroxy[indan-1-spirocyclohexane] detected in UV-C treated leaves [45]. Cinnamic acid amides represented another class of induced compounds, such as cannabisin D, a lignamide resulting from the dimerisation of *N*-*trans*-feruloyltyramine [46], and *N*-*trans*-coumaroyltyramine. The latter has been identified as a wound biomarker and was also up-regulated after the UV-C radiation of *Capsicum annuum* leaves [47] and after herbivore attack in maize leaves [20].

Overall, our untargeted differential metabolomics approach revealed that approximately 15% of the detected features were affected by UV-C radiation. Some of these compounds remain unknown since they are not yet listed in databases and would require more extensive investigations to be identified. For instance, *de novo* induction of oxidised products compounds is expected since UV-C are known to increase ROS levels [10]. This is illustrated by the high level of astringin detected in *Vitis vinifera* which is the oxydised product of piceid. Several of the identified compounds are secondary metabolites with assessed biological activities. For instance, induced cinnamic acid amides in *C. sativa* leaves are known to play a role in cell wall reinforcement after tissue disruption [48]. Stilbenes induced in Vitaceae species contribute to a constitutive defence against microbial diseases [49] and powdery mildew infestation [50]. The antioxidant properties of stilbenes are also well documented [51], and in particular, several studies have shown stronger antiradical scavenging activities for cyclised stilbenes [52].

### 2.5. Antioxidant Activity of Extracts

**Figure 4:**
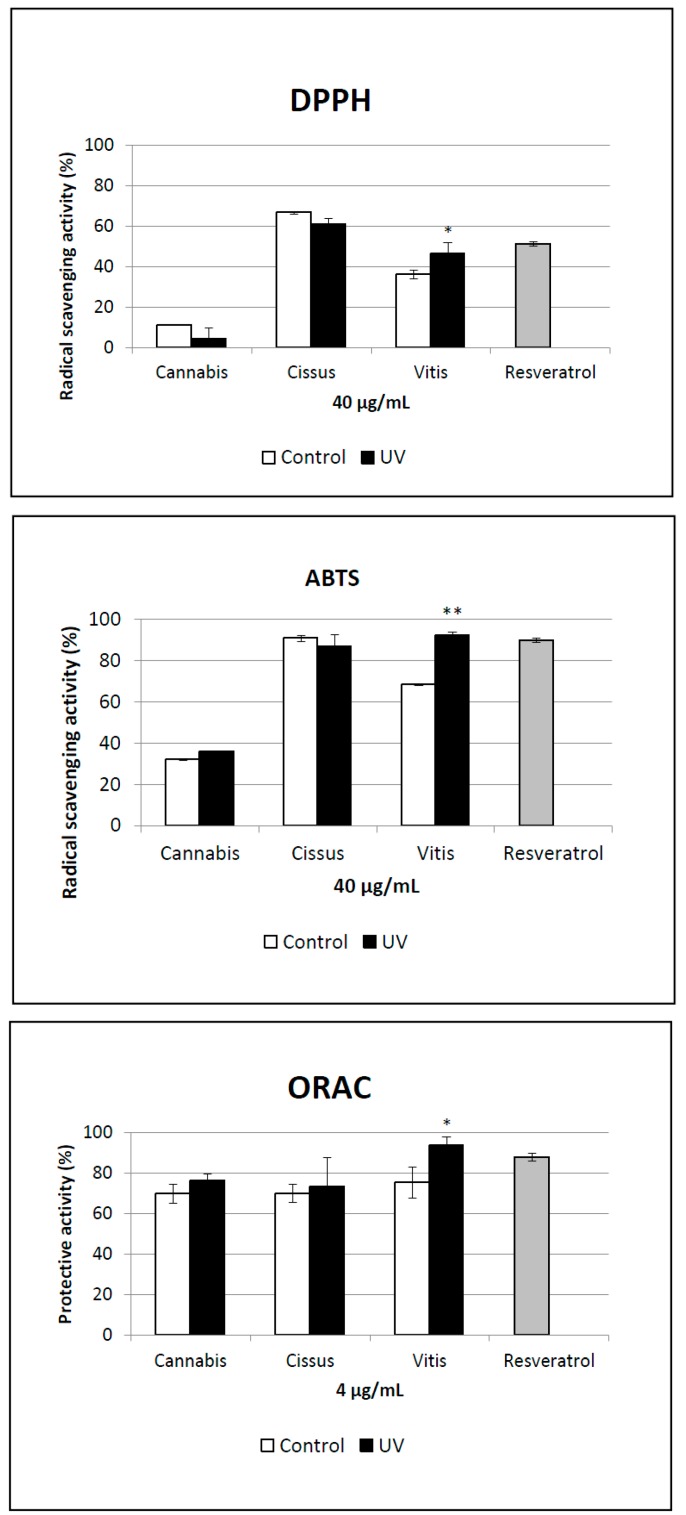
Antioxidant activity of plant extracts compared to resveratrol as positive control. UV indicates plants exposed to UV light, and control indicates non-exposed plants. * Significantly different from control (*p* ≤0.05); ** Significantly different from control (*p* ≤0.001).

**Figure 5:**
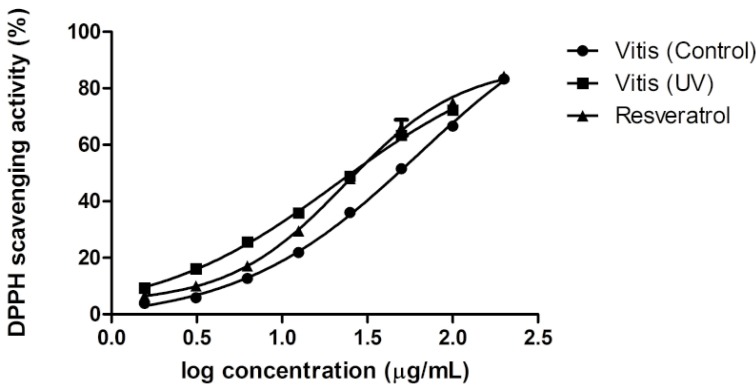
Dose-response curves of the radical scavenging activity on DPPH for *V. vinifera* control, *V. vinifera* UV and resveratrol.

To determine whether the metabolomic modifications observed could be linked to noticeable changes in bioactivity, the antioxidant properties of crude extracts obtained from control and UV-C-treated leaves were assessed. For this analysis, three radical scavenging assays were performed (ABTS, DPPH, and ORAC; see experimental section for details). No activity was found in *C. sativa* extracts, but a significant activity was measured for the *C. antarctica* extracts independently of UV treatment (Figure 4). Interestingly, the radical scavenging activity of the *V. vinifera* UV-C-treated extract was significantly higher compared to the controls in all assays. The EC_50_ values for the radical scavenging activity based on DPPH were determined through dose-response experiments with *V. vinifera* control extract, UV-C-treated *V. vinifera* extract, and resveratrol (Figure 5). The EC_50_ for *V. vinifera* control was 45.9 ± 2.5 µg/mL.

When *V. vinifera* was exposed to UV light, the scavenging potential of the plant extract was significantly higher (*p* ≤ 0.001) with an EC_50_ of 25.12 ± 0.5 µg/mL. This value is similar to the EC_50_ obtained for resveratrol (28.8 ± 1.0 µg/mL). According to the metabolomic variation that was measured in this species, the enhancement of radical scavenging activity in the *V. vinifera* UV-C-treated extract could be explained by its higher level of resveratrol polymers [52]. Moreover, the induced astringin is also known to possess stronger antioxidant activity than resveratrol [53].

## 3. Experimental Section

### 3.1. Plant Growth and UV-C Treatment

Plantlets from the *Vitis vinifera* cultivar Chasselas and *Cissus antarctica* were cultivated in a greenhouse in accordance with the methods described by Pezet *et al.* [54]. Briefly, two-eyes woody cuttings of *Vitis vinifera* cultivar Chasselas and herbaceous cuttings of *Cissus antarctica* were cultivated in a mix of Perlite and potting compost. Liquid fertilizer (Vegesan mega, Hauert, Switzerland) was added weekly, and growing conditions were similar for the two species (20 °C, 70% of relative humidity and a daily photoperiod of 16 h using a sodium lamp at 120 watts/m^2^). Seeds of *Cannabis sativa* (birdseeds produced in Switzerland and distributed by Coop) were sowed and cultivated in a greenhouse under a sodium lamp (400 watts/m^2^). For all species studied, the stage “15 leaves fully developed” was required for further experimentation. All the leaves of each plant were detached and immediately transferred with the abaxial face up into large square Petri dishes (24 cm) containing wet blotting paper (180 g, papyrus, Thalwill, Lausanne, Switzerland). UV-C treatment was performed according to Jean-Denis *et al.* with slight modifications [25]: leaf were exposed during 10 min radiation at 253 nm at 21 °C in the dark. The lamp was placed at 13 cm from the leaves and delivered 0.18 Kj/min (TUV 30W, 92 µW·cm^−2^, Philips, Seynod, France). After UV-C exposure, Petri dishes were sealed and incubated in a growth chamber under alternating light and dark conditions (16 h at 22 °C and 8 h at 18 °C, respectively) for 48 h. The controls consisted of leaves of each plant species that underwent the same treatment lacking the UV-C exposure.

### 3.2. Leaf Extraction

Each sample was ground to a powder using a mortar previously frozen in liquid nitrogen. The frozen powder was weighed (300 mg ± 2 mg), and 1.5 mL of isopropanol was immediately added for metabolite extraction. Samples were vortexed, sonicated in a bath at room temperature (5200 Bransonic, Danbury, CT, USA) for 20 min, vortexed again and centrifuged at 10,000 rpm for 2 min (Hettich mikrolitter D 7200, Buford, GA, USA). The supernatant was recovered, and the extraction procedure was repeated. Each isopropanol extract was dried under vacuum (Genevac HT-4X, Ipswich, UK) and suspended in a mixture of 85:15 (v/v) methanol:water for an SPE C_18_ enrichment procedure (100 mg C18 cartridge Sep-Pack^®^, Waters, Milford, MA, USA) to remove highly non-polar compounds. The filtered extracts were dried and dissolved to 1 mg/mL in 85:15 methanol/water for UHPLC-TOF-MS analysis. This protocol was adapted from Glauser and co-workers [26].

### 3.3. Short LC-MS Profiling

Metabolite analysis was performed on a UPLC-PDA-TOFMS instrument (LCT Premier, Waters) equipped with an electrospray ionisation (ESI) source. The LC-MS fingerprint of each extract was obtained using a short UPLC BEH C18 Acquity column (50 × 1.0 mm i.d., 1.7 µm, Waters). The mobile phase consisted of 0.1% formic acid (FA) in water (phase A) and 0.1% FA in acetonitrile (phase B). The linear gradient program was as follows: 98% A for 0.2 min to 100% B over 4.9 min, held at 100% B for a further 1.1 min, and then returned in 0.1 min to initial conditions (98% A) for 1.1 min of equilibration before the subsequent analysis. The flow rate was 0.3 mL/min. The column temperature was kept at 40 °C. Detection was performed by TOF-MS in W-mode in both electrospray (ESI) negative (NI) and positive ion (PI) modes in independent runs with the following settings: capillary voltage at 2.8 kV, cone voltage at 40 V, desolvation temperature at 250 °C, source temperature at 120 °C and desolvation gas flow at 600 L/h. The *m/z* range was 100–1000 Da with a scan time of 0.25 s. The MS was calibrated using sodium formate, and leucine enkephalin was used as an internal reference. The injection volume was 1 µL.

### 3.4. Data Processing and Data Analysis

The UHPLC-TOF-MS fingerprints were processed with MZmine 2.10 for mass signal extraction and alignment from 0 to 5 min with *m/z* values ranging from 100 to 1000 Da. The following parameters were employed: the chromatogram builder was set to a minimum time span of 0.06 min, a minimum height of 10 for NI mode and 100 for PI mode, and an *m/z* tolerance of 10 ppm. The local minimum search algorithm was applied for chromatogram deconvolution. Each peak list was de-isotoped and aligned using the RANSAC alignment method and then gap-filled. The resulting peak matrix from each sample containing areas of aligned peaks characterised by retention time and *m/z* ratio was exported into the “.csv” file-format prior to multivariate data analysis using SIMCA-P+ (version 12, Umetrics, Umeå, Sweden). Homemade Excel macros were used to compare data between PI and NI modes and between treated and control samples, and *p*-values were calculated by Student’s T-test. 

### 3.5. Standards

*N*-*p*-*trans-*coumaroyltyramine (NMR purity of 99%) was purified from *Zea mays* leaves according to a procedure described previously [55]. Resveratrol (GC purity ≥ 99%) and piceid (>95% HPLC) were purchased from Sigma-Aldrich (Sigma-Aldrich Chemie GmbH, Buchs, Switzerland).

### 3.6. Antioxidant Assays

DPPH radical scavenging assay: the capacity of samples to scavenge the stable radical 2,2-diphenyl-1-picrylhydrazyl (DPPH) was determined spectrophotometrically by measuring the loss of absorbance of DPPH at 515 nm [56]. Clear polystyrene flat-bottom 96-well microplates were filled with test sample solution (in ethanol containing up to 2% DMSO) or vehicle for the DPPH control. The reaction was initiated by the addition of 80 µM DPPH (in ethanol). The decrease in absorbance at 515 nm was monitored at room temperature after 10 min to determine the percentage of scavenged radical. Samples were tested in triplicate at 40 µg/mL, and dose-response experiments were performed with at least 6 concentrations.

ABTS radical scavenging assay: the capacity of samples to scavenge the monocation radical 2,2'-azinobis-(3-ethylbenzothiazoline-6-sulfonic acid) (ABTS) was determined spectrophotometrically by measuring the loss of absorbance of ABTS at 715 nm [57]. The same procedure as described for the DPPH assay was applied by using the ABTS solution instead of DPPH solution. The ABTS cation was produced by the reaction between 7 mM ABTS and 2.45 mM potassium persulfate in water. The reaction was initiated by the addition of 67 µM ABTS radical (in ethanol). Samples were tested in triplicate at the same concentrations used for the DPPH assay.

ORAC assay: the antioxidant activity of the tested samples was determined by their ability to preserve the fluorescence of fluorescein exposed to peroxyl radicals generated by 2,2'-azobis(2-methylpropionamidine) dihydrochloride (AAPH) [58]. Black polypropylene 96-well plates were filled with 60 nM fluorescein (in glycine buffer pH 8.3), together with test samples or vehicle (in 2% DMSO), and pre-incubated at 40 °C for 15 min. The oxidative reaction was obtained by adding 5 mM AAPH (in glycine buffer pH 8.3) to wells containing samples, positive control, and oxidised fluorescein control. Non-oxidised fluorescein controls were added with the same volume of assay buffer. The plate was incubated at 40 °C for 90 min with continuous shaking at 150 rpm and cooled to room temperature (5 min) prior to fluorescence reading at 485/528 nm. Samples were tested in triplicate at 4 µg/mL.

## 4. Conclusions

This LC-HRMS-based metabolomic study revealed important metabolic changes upon the UV-C treatment of the leaves of three different plant species. The metabolomic modifications were found to be species-specific, but in all plants studied, the UV-C stress significantly induced greater than five-fold changes for more than 10% of the features detected. The LC-MS-based approach used provided a holistic overview of the changes related to a given stress and generated useful data for a rapid estimation of the magnitude of UV-C-induced metabolomic modifications and a preliminary identification of related biomarkers.

In the case of *C. sativa*, no remarkable modification of the cannabinoid content was observed, but dehydrostilbenes and cinnamic acid amide derivatives were strongly induced. In contrast, Vitaceae species responded in the same manner with a strong induction of stilbenes derived from resveratrol. Furthermore, it has been demonstrated that in some cases (e.g., for *V. vinifera*) such metabolome modifications could enhance the antioxidant activity of the extracts.

This generic approach may represent an interesting method to screen leaves for new bioactivities or new metabolites that would not be detected without stress induction (e.g., stilbene polymers from Vitaceae species) and that may be related to cryptic biosynthetic pathways. Because common metabolites could be observed after UV-C stress treatment and other biotic or abiotic stresses (e.g., wound response, pathogen infection, *etc.*), such methods can also be used to indicate the overall metabolite induction potential of a given plant compared to more specific and relevant biological stresses.
